# A new live-cell reporter strategy to simultaneously monitor mitochondrial biogenesis and morphology

**DOI:** 10.1038/srep17217

**Published:** 2015-11-24

**Authors:** Linn Iren Hodneland Nilsson, Ina Katrine Nitschke Pettersen, Julie Nikolaisen, David Micklem, Hege Avsnes Dale, Gro Vatne Røsland, James Lorens, Karl Johan Tronstad

**Affiliations:** 1 Department of Biomedicine, University of Bergen, Bergen, Norway; 2 BerGenBio AS, Bergen, Norway; 3 Molecular Imaging Center, Department of Biomedicine, University of Bergen, Bergen, Norway

## Abstract

Changes in mitochondrial amount and shape are intimately linked to maintenance of cell homeostasis via adaptation of vital functions. Here, we developed a new live-cell reporter strategy to simultaneously monitor mitochondrial biogenesis and morphology. This was achieved by making a genetic reporter construct where a master regulator of mitochondrial biogenesis, nuclear respiratory factor 1 (NRF-1), controls expression of mitochondria targeted green fluorescent protein (mitoGFP). HeLa cells with the reporter construct demonstrated inducible expression of mitoGFP upon activation of AMP-dependent protein kinase (AMPK) with AICAR. We established stable reporter cells where the mitoGFP reporter activity corresponded with mitochondrial biogenesis both in magnitude and kinetics, as confirmed by biochemical markers and confocal microscopy. Quantitative 3D image analysis confirmed accordant increase in mitochondrial biomass, in addition to filament/network promoting and protecting effects on mitochondrial morphology, after treatment with AICAR. The level of mitoGFP reversed upon removal of AICAR, in parallel with decrease in mtDNA. In summary, we here present a new GFP-based genetic reporter strategy to study mitochondrial regulation and dynamics in living cells. This combinatorial reporter concept can readily be transferred to other cell models and contexts to address specific physiological mechanisms.

Mitochondria are energetic and metabolic headquarters in the cell. This role is tightly associated with their tasks as stress sensors and mediators in processes such as adaptation, autophagy, and cell death^1^,^2^,^3^. The ability to control and maintain mitochondrial biomass and functional quality is therefore essential in cell (patho)physiology, and has been linked to conditions such as diabetes, neurodegeneration and cancer^4^. Adjustments, or defects, in mitochondrial functions are often accompanied by changes in organelle biomass and morphology (i.e. mitochondrial dynamics) (reviewed in^5^,^6^). To this end, mitochondrial biogenesis is crucial to prevent cellular stress by balancing changes in energy demand and replenishing damaged mitochondria^7^. In order to understand more about the physiological cues controlling context-dependent mitochondrial adjustments, we need methods that integrate regulatory and structural aspects of these organelles in living cells. In the present study we combined genetic reporter tools to monitor transcriptional activity with organelle-specific localisation of the fluorescent reporter protein, to simultaneously assess mitochondrial biogenesis and morphology. This proved to be a promising conceptual strategy to study mitochondrial adaptations in living cells.

The mitochondrion is a double membrane organelle that contains multiple copies of a small circular DNA molecule (mtDNA). These organelles house many metabolic pathways, both catabolic and anabolic, and account for a major part of the cellular ATP production via oxidative phosphorylation (OXPHOS) (6and references therein). In the OXPHOS process, the mitochondrial membrane potential is created by transmembrane proton transport from the matrix compartment, driven by electron transport through the OXPHOS protein complexes I–IV. Subsequently, reverse proton flow powers ATP synthesis by the action of ATP synthase (OXPHOS complex V). Complex IV consumes molecular oxygen as terminal electron acceptor (i.e. mitochondrial respiration), and analysis of oxygen consumption can therefore be used to measure OXPHOS rates (e.g.^8^,^9^). The standard conception is that rates of mitochondrial respiration correlate with the amount of mitochondrial biomass in the cell; however, mitochondrial integrity and respiratory function may change depending on cellular conditions and incidents. Such effects may also involve characteristic changes in mitochondrial morphology and dynamics^5^.

The functional purpose of mitochondrial biogenesis is to maintain mitochondrial quality and secure sufficient ATP production^10^,^11^. Gene transcription and mtDNA replication are crucial in this process, to provide building blocks for new mitochondria. Crosstalk between the nuclear and mitochondrial genomes is therefore required to coordinate the synthesis of new organelles^12^,^13^. The transcription factor nuclear respiratory factor 1 (NRF-1) is essential in this regard, since it regulates the expression of multiple mitochondrial proteins encoded by nuclear genes. NRF-1 was initially characterised as an activator of cytochrome *c* expression^14^, and was subsequently found to regulate expression of additional OXPHOS subunits (reviewed in^15^). NRF-1 is now established as a master regulator of mitochondrial biogenesis (reviewed in^6^). One of the major routes of NRF-1 activation is via the cellular energy sensor AMP-activated protein kinase (AMPK) (reviewed in^16^). AMPK is activated by increased levels of AMP, i.e. energy depletion, and leads to expression of the peroxisome proliferator-activated receptor γ coactivator-1α (PGC-1α), which co-activates NRF-1^17^. This results in transactivation of NRF-1 target genes, including mitochondrial transcription factor A (TFAM)^18^. Activation of AMPK with 5-amino-1-β-D-ribofuranosyl-imidazole-4-carboxamide (AICAR) is known to stimulate mitochondrial biogenesis in many cell types, including HeLa cells^19^,^20^,^21^. AICAR acts by trigging phosphorylation of AMPK^22^, which typically leads to activation of energy yielding processes, and inhibition of energy requiring processes, in the cell^16^.

Fluorescence microscopy and quantitative image analysis represent important tools in mitochondrial research^6^,^23^. Mitochondria are then visualised in intact/living cells using chemical probes or expression of mitochondria targeted fluorescent proteins (e.g. GFP). Following image acquisition, quantitative analysis facilitates extraction of multiple mitochondrial parameters in 2 or 3 dimensions (2D, 3D), depending on the specimen/cell type^24^,^25^. In the present study, we imaged mitochondria in cells expressing GFP with a mitochondrial localisation sequence (mitoGFP) as a reporter for NRF-1 activity. The reporter construct was produced by combining the promoter region of an already established NRF-1 luciferase reporter^26^ with the gene for mitoGFP. As mitoGFP (and not luciferase) can be detected in living cells, this approach fulfilled the objective to enable real-time studies of mitochondrial regulation in living cells. In conclusion, we developed a novel live-cell reporter system for simultaneous analysis of AMPK/NRF-1-regulated gene transcription, and the subsequent effects on mitochondrial biomass and morphology.

## Results

### Generation of stable NRF-1 reporter cells with dynamic expression of mitoGFP

**Figure 1:**
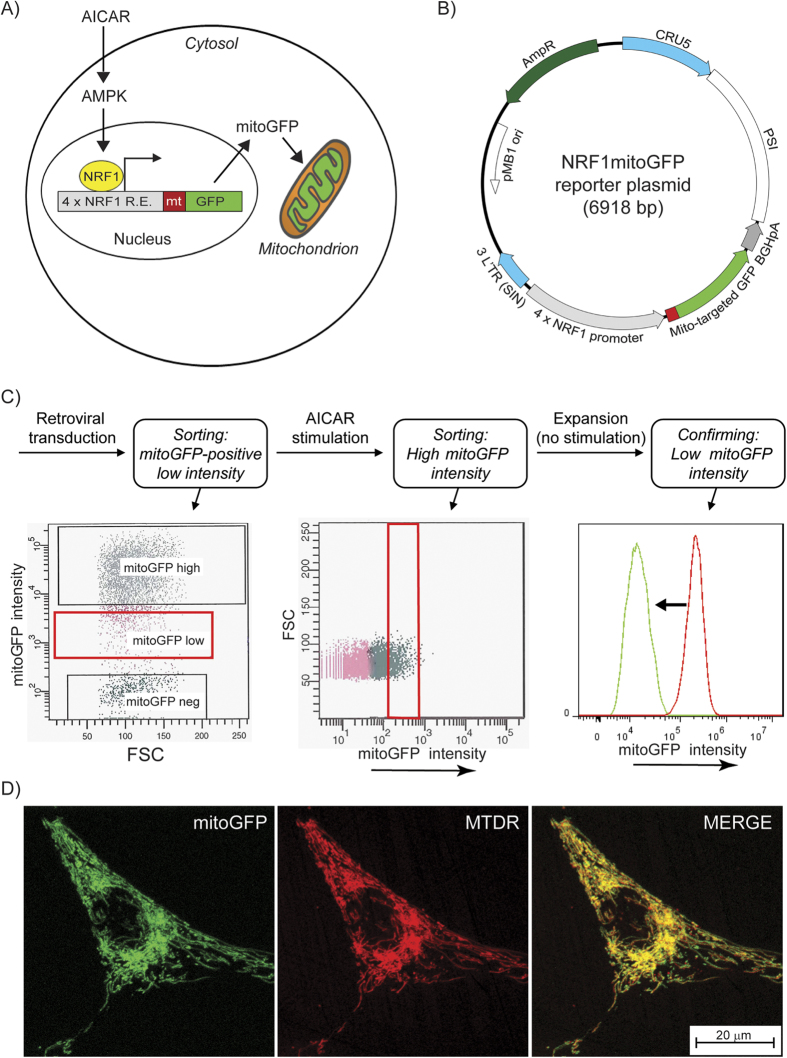
The live-cell mitochondrial reporter model. (**A**) Overview of the live-cell mitochondrial reporter model. A reporter construct (NRF1mitoGFP) with mitoGFP under the control of a promoter with NRF-1 responsive elements (R.E.) was inserted in HeLa cells. AICAR was used to trigger NRF-1 reporter activity, via AMPK. The cellular expression of mitoGFP (intensity) reflected transcriptional responses of mitochondrial biogenesis, whereas the mitochondrial localisation of the mitoGFP protein enabled quantitative image analysis of mitochondrial morphology and biomass. (**B**) NRF1mitoGFP construct map. (**C**) Establishment of reporter cells. Flow cytometry was used to sort responding cells based on mitoGFP expression, depending on the presence of AICAR. The figure shows the stepwise strategy to isolate cells with inducible and reversible mitoGFP expression after treatment with AICAR (0.5 mM). The red gates in the two dot plots show the sorted populations. The histogram shows how the mitoGFP intensity shifted from high (red curve) to low (green curve) when AICAR was removed from the reporter cells. (**D**) Colocalisation of mitoGFP and MitoTracker Deep Red (MTDR) in HeLaNRF1/c4 cells after 6 days treatment with AICAR (0.5 mM). The images show contrast corrected maximum intensity composites (MICs) of a representative cell (imaged on a Zeiss LSM 510 META confocal microscope).

In order to establish a live-cell reporter system for assessment of mitochondrial biogenesis and morphology (Fig. 1A), we made a genetic reporter construct (NRF1mitoGFP) expressing mitochondrial targeted GFP under the control of a NRF-1 regulated promoter (Fig. 1B). Based on the knowledge that AMPK induces mitochondrial biogenesis via activation of NRF-1^27^, the AMPK activator AICAR was used to induce reporter activity. The construct was stably inserted into HeLa cells by retroviral transduction, and cells that responded to AICAR treatment by expressing high mitoGFP intensity were sorted (Fig. 1C). After clonal expansion, each clone were tested by repeated stimulation (AICAR) - rest cycles, followed by sorting of cells with high and low mitoGFP intensities, respectively. In additional experiments, sub-populations of responding cells were isolated instead of single cells (data not shown). These sub-populations reported similar data as the isolated clones, which confirms that this reporter also may be employed without clonal isolation. Cells with an identical reporter construct lacking the NRF-1 response element (-66mitoGFP) did not demonstrate AICAR-dependent mitoGFP expression (data not shown). We isolated four clones demonstrating sustainable and reproducible AMPK/NRF-1 regulated response depending on the presence of AICAR; one of these was selected for further validation and characterisation of mitochondrial dynamics. This clone is referred to as HeLaNRF1/c4. Microscopy confirmed that the mitoGFP colocalised with MitoTracker Deep Red in the mitochondrial network (Fig. 1D). The mitoGFP fluorescence was found to distribute evenly throughout the mitochondrial compartment, which is a crucial advantage for fluorescence-based image analysis of mitochondrial shape.

### Markers of mitochondrial biogenesis

**Figure 2:**
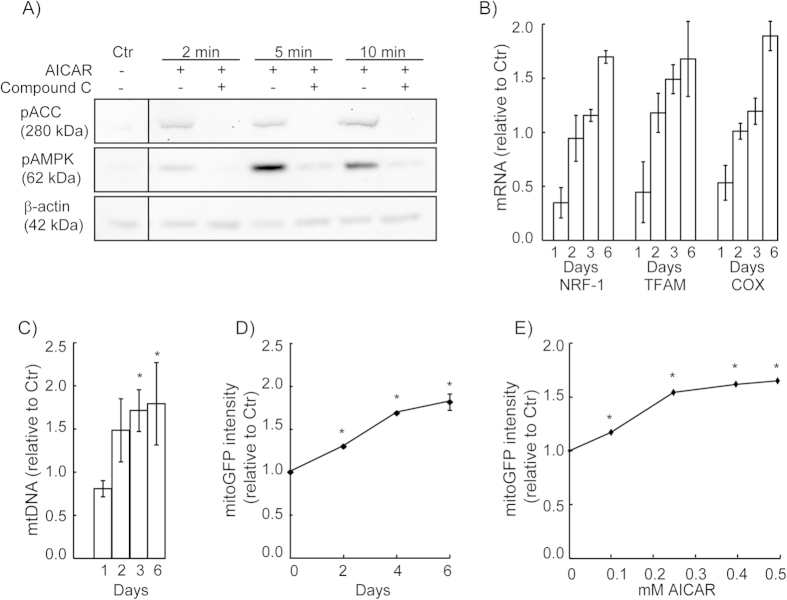
Biochemical markers of mitochondrial biogenesis in the reporter cells. (**A**) AMPK activation in HeLaNRF1/c4 cells. The cells were treated with 0.5 mM AICAR for 2, 5 or 10 min, with or without the AMPK inhibitor Compound C (10 μM). The levels of phosphorylated AMPK (pAMPK) and ACC (pACC) were analysed by western blotting. (**B**) The effect of AICAR (0.5 mM) on expression of NRF-1, TFAM and COX was measured at four different time points (1, 2, 3 and 6 days), by quantitative PCR. The data were calculated relative to the untreated control (Ctr) and are shown as mean ± S.D. of two experiments with triplicate measurements. (**C**) The amount of mtDNA relative to nuclear DNA (mtDNA copy number) was measured by quantitative PCR, in cells grown for 1, 2, 3 or 6 days in presence of AICAR (0.5 mM). The data were calculated relative to untreated control (Ctr), and are shown as mean ± S.D. of three experiments with triplicate measurements. (**D**) Kinetics of mitoGFP induction. The expression of mitoGFP was measured by flow cytometry at different time points during 6 days of treatment with AICAR (0.5 mM). The data represents mean ± S.D. of three experiments (10 000 cells per sample), calculated relative to untreated cells. (**E**) Dose-response of AICAR in HeLaNRF1/c4 cells. The cells were treated with increasing concentrations of AICAR for 6 days and mitoGFP was detected by flow cytometry (10 000 cells per sample). The data represent mean ± S.D. of n ≥ 3. *p < 0.05; (**C**) compared with day 1, ANOVA test with Tukey’s multiple comparisons test; (**D, E**) compared with Ctr, Student’s t-Test.

In order to confirm that AICAR activates AMPK in the HeLaNRF1/c4 cells, we analysed phosphorylation of AMPK and its downstream target acetyl-CoA carboxylase (ACC) by western blotting. Phosphorylation of both AMPK and ACC was initiated already after two minutes of AICAR treatment, and increased after 5 and 10 minutes (Fig. 2A). The phosphorylation was blocked by the presence of the specific AMPK inhibitor, Compound C. To document that AMPK activation induced NRF-1-regulated mitochondrial biogenesis in these reporter cells, we measured the mRNA levels of NRF-1 and two of its target genes, cytochrome *c* oxidase (COX) and TFAM, in a time-lapse experiment (Fig. 2B). Following an initial decline in all mRNAs compared to control (24h), the levels steadily increased during the period of 2–6 days. In parallel measurements, the amount of mtDNA relative to nuclear DNA (i.e. mtDNA copy number) increased in accordance with the other markers of mitochondrial biogenesis (Fig. 2C). The effects of AICAR on gene expression and mtDNA coincided with a corresponding increase in mitoGFP expression in the reporter cells (Fig. 2D). A dose-response experiment showed that 0.5 mM AICAR gave maximum mitoGFP response in the reporter cells, and was well tolerated (Fig. 2E). These data confirm that AICAR triggers the normal response of AMPK/NRF-1-regulated mitochondrial biogenesis, and that mitoGFP expression reports the activation status of AMPK/NRF-1-regulated mitochondrial biogenesis in the HeLaNRF1/c4 reporter cells.

### Quantitative image analysis of mitochondria in single cells

**Figure 3:**
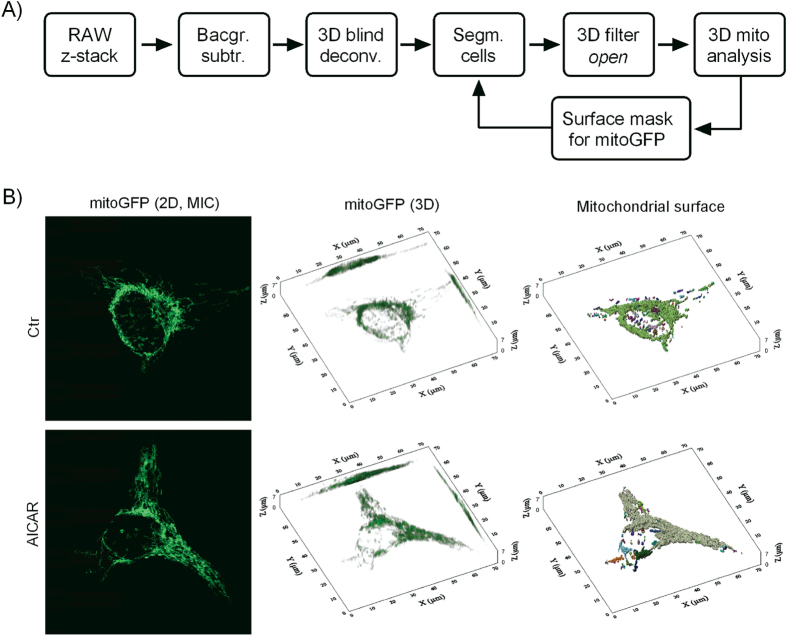
Quantitative 3D image analysis of mitochondrial morphology. (**A**) Image processing and analysis was performed essentially as described previously^25^. The illustration gives an overview over the individual steps of the procedure. Background correction was performed on the unprocessed confocal z-stacks (RAW), by subtracting a fixed intensity decided from non-mitochondrial areas. Following 3D blind deconvolution, individual cells were segmented manually to enable single-cell analysis. The resulting z-stacks were processed by the 3D open filter, and loaded into the 3D module for analysis. The isosurface created in this process was applied as mask for measurement of mitoGFP intensity in the mitochondrial compartment, in the respective z-stacks not processed by the 3D open filter. B) Mitochondria imaged in untreated (Ctr) and AICAR-treated HeLaNRF1/c4 cells. The left-hand images show 2D maximum intensity composites (MICs) of the processed z-stacks from two representative cells. The centre images show the same cells in 3D, based on mitoGFP from all the z-stack section. The right-hand images show the mitochondrial isosurface, which was created to segment and analyse the mitochondria.

**Figure 4:**
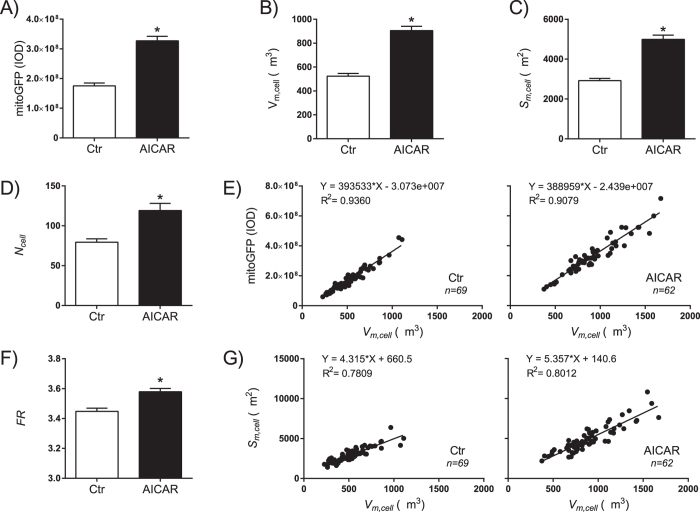
Quantitative analysis of mitochondrial biomass in single cells. Confocal microscopy, and quantitative image analysis of mitoGFP was performed on untreated (Ctr) and AICAR-treated (0.5 mM, 6 days) HeLaNRF1/c4 cells. Data were extracted from analysis of single cells (Ctr, n = 69; AICAR, n = 62) and are shown as and mean ± SEM. (**A**) Total mitoGFP intensity in the mitochondrial compartment, represented by the Integrated Optical Density (IOD). (**B**) Mitochondrial volume per cell (*V*_*m,cell*_). (**C**) Mitochondrial surface area per cell (*S*_*m,cell*_). (**D**) Number of mitochondrial objects per cell (*N*_*cell*_). (**E**) Linear regression analysis of *V*_*m,cell*_ vs mitoGFP. Each dot represents a single cell. (**F**) Average Feret ratio (*FR*) of mitochondrial objects. (**G**) Linear regression analysis of *V*_*m,cell*_ vs *S*_*m,cell*_. *p < 0.0001 compared with Ctr.

Confocal microscopy and image-based mitochondrial analysis^25^ was used to quantify effects of AMPK/NRF-1 activation on mitochondrial biomass and morphology in the HeLaNRF1/c4 reporter cells. The z-stacks acquired by confocal microscopy were processed and segmented for single cell analysis (Fig. 3). The data demonstrated that the AICAR-treated cells had significantly increased level of mitochondrial fluorescence from mitoGFP (Fig. 4A), and this coincided with increased cellular quantities of mitochondrial volume (*V*_*m,cell*_), surface area (*S*_*m,cell*_), and number (*N*_*cell*_) (Fig. 4B–D). A minor fraction of the AICAR-treated cells had relatively low scores of mitoGFP and mitochondrial volume, most likely representing a small sub-population of non-responding cells which was also observed by flow cytometry (data not shown). Regression analysis revealed a strong correlation between the cellular mitoGFP intensity and *V*_*m,cell*_, which was equivalent (i.e. the slope) in control and AICAR-treated cells (Fig. 4E). This observation warrants the use of total cell mitoGFP fluorescence (e.g. by flow cytometry) as a readout for mitochondrial biomass in these reporter cells. To study possible effects on the filamentous character of the mitochondria in these cells, we measured the Feret Ratio (*FR*), which is the ratio between the object length and width. The data demonstrated a small, but significant increase in the mean *FR* in AICAR-treated cells, indicating that the filamentous character is strengthened. Furthermore, a morphological change was substantiated by the change in the correlation between *S*_*m,cell*_ and *V*_*m,cell*_ (Fig. 4G) in the AICAR-treated cells. The increased slope of the regression line demonstrated a shift towards more surface area per volume, which corresponds well with extension of filamentous structures.

### Quantitative image analysis of mitochondrial morphology

**Figure 5:**
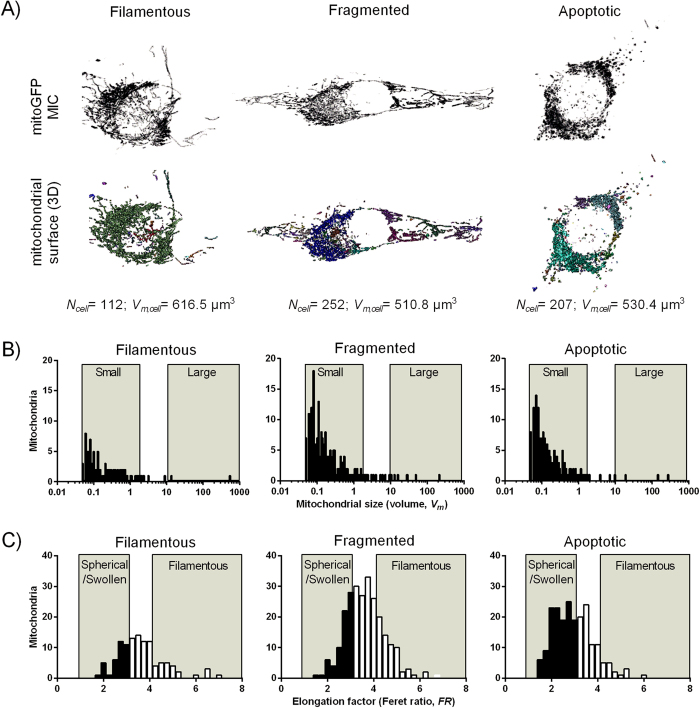
Assessment of mitochondrial morphology. HeLaNRF1/c4 cells were treated with CCCP (5 μM, 30–60 min) to induce mitochondrial fragmentation. Mitochondrial fragmentation was observed in most cells, and some cells (very few) showed signs of apoptotic mitochondrial morphology. (**A**) Three cells with similar amount of mitochondrial biomass (*V*_*m,cell*_), but with distinct mitochondrial morphologies, were selected to characterize the different mitochondrial subpopulations. The three types of morphology are shown as “filamentous”, “fragmented”, and “apoptotic”. The upper images show contrast enhanced projections (mitoGFP, MIC) of the z-stacks, with the surface of the respective 3D-segmented mitochondrial objects underneath. (**B**) Frequency distribution analysis based on *V*_*m*_. The following mitochondrial subpopulations were defined: “small”, *V*_*m*_ = 0.05–2 μm^3^; “medium size” *V*_*m*_ = 2–10 μm^3^; “large”, *V*_*m*_ > 10 μm^3^. The gates of the “small” and “large” subpopulations are shown in the diagram. (**C**) Frequency distribution analysis based on Feret ratio (*FR*). The following mitochondrial subpopulations were defined: “spherical/swollen”, *FR* = 1–3; “elongated”, *FR* = 3–4; “filamentous”, *FR* > 4). The gates of the “spherical/swollen” and “filamentous” subpopulations are shown in the diagram.

**Figure 6:**
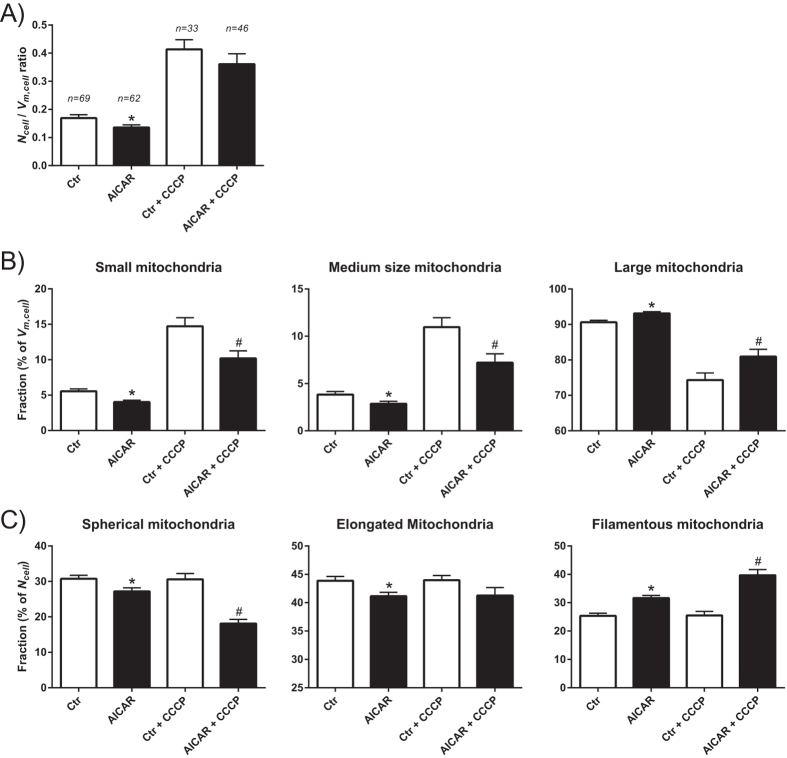
Quantitative analysis of mitochondrial dynamics. Untreated (Ctr) and AICAR-treated (0.5 mM, 6 days) HeLaNRF1/c4 cells were imaged as described in Fig. 4. CCCP (5 μM, 30–60 min) was added to induce mitochondrial fragmentation. Data were extracted from analysis of single cells and are shown as and mean ± SEM. (**A**) Number of mitochondria relative to biomass (*N*_*cell*_/*V*_*m,cell*_ ratio). The number of cells analysed for each treatment is shown. (**B**) The relative amount (percentage) of the mitochondrial biomass (volume) found in the three different size-populations. (**C**) The relative number (percentage) of the mitochondria found in the three different shape-populations. *p < 0.05 compared with Ctr, ^#^p < 0.05 compared with Ctr+CCCP.

Based on effects on the cellular level in the AICAR-treated reporter cells, we analysed single mitochondria to identify and characterise the properties of mitochondrial subpopulations. In these studies, we included treatment with the mitochondrial uncoupler carbonyl cyanide m-chlorophenylhydrazone (CCCP), to characterise the effects of mitochondrial fragmentation on the analytical output^28^. From these experiments, we first selected the confocal z-stacks from three cells with distinct mitochondrial morphologies, namely filamentous, fragmented and apoptotic (Fig. 5A). The data from these cells were employed to establish a strategy to analyse mitochondrial subpopulations based on single organelle volume (*V*_*m*_) and elongation (*FR*) (Fig. 5A,B). The following two types of subpopulation classification were defined: I) size-populations (small, *V*_*m*_ = 0.05–2 μm^3^; medium size *V*_*m*_ = 2–10 μm^3^; large, *V*_*m*_ > 10 μm^3^), and II) shape-populations (spherical/swollen, *FR* = 1–3; elongated, *FR* = 3–4; filamentous, *FR* > 4). Accordingly, the cell with filamentous mitochondria contained relatively few organelles, with a dominant compartment constituting most of the mitochondrial volume in the cell (Fig. 5B). In contrast, the cell with fragmented mitochondria contained significantly increased number of organelles, particularly in the population of small mitochondria. This was similar in the apoptotic cell; however, this cell had an increased fraction of spherical or swollen mitochondria compared to the other two (Fig. 5C). These data were in accordance with the visual characterization of the mitochondria, and confirmed that this method is adequate for assessing different types of mitochondrial morphology in a quantitative manner. The subsequent analysis of the entire dataset revealed that AICAR-treated cells had a modest, but significant, reduction in mitochondrial number relative to total biomass (*N*_*cell*_/V_*m,cell*_) (Fig. 6A). Treatment with CCCP induced a major increase in mitochondrial number in both control and AICAR-treated cells. Further subpopulation analysis showed that AICAR treatment decreased the relative fractions of small and medium size organelles, whereas the fraction of large organelles was increased (Fig. 6B). Treatment with CCCP clearly increased the fraction of small and medium sized organelles, and reduced the fraction of large mitochondria, which is in accordance with mitochondrial fragmentation. Apparently, the effects of CCCP were reduced in the AICAR-treated cells compared to control cells. Furthermore, the AICAR-treated cells had an increased subpopulation of filamentous mitochondria, whereas the fraction of spherical and elongated organelles were reduced (Fig. 6C). Interestingly, CCCP caused a significant decrease in the fraction of spherical mitochondria, accompanied by an increase in filamentous organelles, exclusively in the AICAR-treated cells. This is probably explained by the findings that these cells have a more extensive filamentous network, which may produce a larger relative quantity of filamentous mitochondrial fragments which dilute the subpopulation of spherical mitochondria, as observed in these data. In summary, these data demonstrate that AICAR-treatment strengthen filamentous network characters of the mitochondria, and reduces the amount of small organelles. Furthermore, this seems to mediate protective effects on the morphological changes caused by CCCP.

### OXPHOS protein expression and function

**Figure 7:**
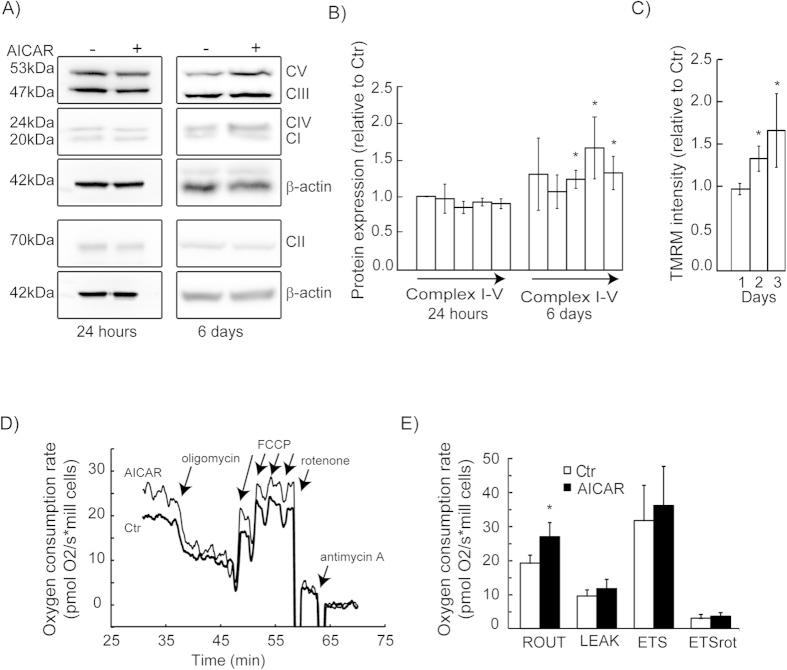
Mitochondrial respiration. (**A**) Western blot analysis of OXPHOS proteins of complex I–V (CI-V), in HeLaNRF1/c4 cells treated with 0.5 mM AICAR for 24 hours or 6 days. The shown blots are representative for three independent experiments. (**B**) Data quantified from the immunoblots (in **A**). Protein expression levels (normalised to β-actin) were calculated relative to untreated control (Ctr) and are shown as mean ± S.D. of three experiments. (**C**) Mitochondrial membrane potential was studied using the fluorescent probe TMRM, after 1, 2 and 3 days of treatment with AICAR (0.5 mM). The data represents mean ± S.D. of three experiments (10 000 cells per sample). (**D, E**) Oxygen consumption rates in HeLaNRF1/c4 cells after treatment with AICAR (0.5 mM, 6 days). (**D**) The diagram shows a representative experiment. (**E**) Data analysis of routine (basal) respiration rate (ROUT), uncoupled respiration (LEAK), respiratory capacity (ETS), and rotenone insensitive respiration (ETSrot). Data are presented as mean ± S.D. of five experiments. *p < 0.05 comparing AICAR-treated cells with Ctr.

To determine if activation of AMPK/NRF-1-regulated mitochondrial biogenesis includes expression of OXPHOS machinery in the HeLaNRF1/c4 reporter cells, we measured selected subunits by western blotting after treatment with AICAR (Fig. 7A). Densiometric quantification of the immunoblots showed increased expression of the subunits of OXPHOS complex III, IV and V (ATP synthase) (Fig. 7B). The expression of complex I tended to increase, but this was not statistically significant. In an additional experiment, the effects on OXPHOS expression were equal in the HeLaNRF1/c4 reporter cells compared with non-modified HeLa cells, after treatment with AICAR (data not shown). This confirmed that the reporter cells have conserved the original physiological response.

To evaluate how the increase in mitochondrial biomass and OXPHOS machinery affects the bioenergetic capacity, we first performed flow cytometry to measure cellular accumulation of TMRM. A gradual increase in cellular TMRM was observed in the AICAR-treated cells during the three days of the experiment (Fig. 7C). This effect is most likely due to increased mitochondrial biomass in the treated cells, and not hyperpolarisation of the membrane potential. Oxygen consumption measurements confirmed that the treated cells had a significantly increased mitochondrial respiration (40%) (Fig. 7D,E). Subsequent injection of oligomycin (ATP synthase inhibitor) and FCCP (uncoupler) confirmed that membrane integrity and respiratory control were intact. The maximal rate of the electron transport system, determined after complete uncoupling with FCCP, showed a tendency of increased respiratory capacity, however, this effect was not statistically significant. Mitochondrial respiration was primarily driven by OXPHOS complex I, since addition of rotenone, an inhibitor of this complex, nearly eliminated oxygen consumption. In summary, induction of mitochondrial biogenesis was associated with stimulation of mitochondrial respiration under normal cell culture conditions.

### Reversion of mitochondrial adaptations

**Figure 8:**
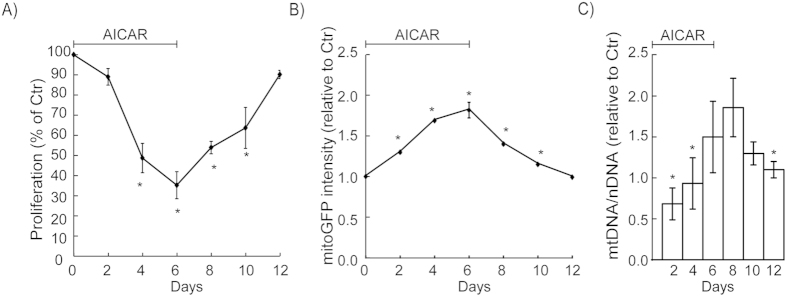
Mitochondrial reversion after withdrawal of AICAR. HeLaNRF1/c4 cells were cultured in presence of AICAR (0.5 mM) for 6 days, followed by a subsequent period (6 days) in absence of AICAR. Samples were collected and analysed every second day during the experimental period. (**A**) Proliferation was examined by cell counting. The data, calculated as percent of the untreated control cultures (Ctr), are shown as mean ± S.D. of three independent experiments. (**B**) The intensity of mitoGFP was detected by flow cytometry (10 000 cells/sample). The diagrams show the mitoGFP level relative to control as mean ± S.D. of three independent experiments. (**C**) mtDNA (copy number) determined by quantitative PCR. Data are presented as mean ± S.D. of six independent experiments with triplicate measurements. *p < 0.05; (**A, B**) compared with Ctr, Student’s t-Test; (**C**) compared with 8 days, ANOVA test with Tukey’s multiple comparisons test.

In order to evaluate the physiological relevance of our reporter model it was important to determine if the responses were reversible when the stimulus (AICAR) was withdrawn. We therefore treated the cells for 6 days with AICAR, and then changed to medium without AICAR for 6 days. Treatment with AICAR reduced cell proliferation (Fig. 6A), which agrees with engagement of energy-saving programs controlled by AMPK^16^. Subsequent removal of AICAR led to re-establishment of cell growth, which confirms that the physiological adaptations were reversible. Correspondingly, the AICAR-induced increase in mitoGFP (Fig. 8B) and mtDNA (Fig. 8C) reversed when the treatment was terminated. The effects on mitoGFP and mtDNA were similar in magnitude and kinetics; however, the maximum mtDNA level was seen 2 days after end of treatment, and later than for mitoGFP. The delay is most likely explained by the fact that mtDNA replication occurs downstream of transcriptional regulation, which is reported by mitoGFP. Both mitoGFP and mtDNA had returned to normal levels 6 days after ending the treatment. Hence, these data suggest the half-life of AICAR-induced mitoGFP is approximately 3 days under these conditions. In summary, this experiment confirms that the cellular and mitochondrial effects of AICAR are reversible, and that the level of mitoGFP reports a relevant and valid measure of mitochondrial biogenesis.

## Discussion

The presented work describes a new GFP-based reporter approach for multifaceted studies of mitochondrial regulation in living/intact cells. The method enabled surveillance of the AMPK/NRF-1–controlled program of mitochondrial biogenesis, simultaneous with quantitative analysis of mitochondrial biomass and morphology. Expression of the mitoGFP reporter was activated with AICAR, and correlated with markers of NRF-1 transactivation and mitochondrial biogenesis, both in magnitude and kinetics (1.5–2 fold after 6 days). Confocal microscopy and quantitative 3D image analysis confirmed corresponding effects on mitochondrial biomass and morphology. The induction of mitochondrial biogenesis via AMPK/NRF-1 was reversible and compatible with long term cell survival.

Based on the presented results, the NRF1mitoGFP reporter plasmid was found to facilitate simultaneous analysis of NRF-1 activity and mitochondrial morphology in intact/living cells. The construct was stably inserted into HeLa cells, and clones (or populations) that demonstrated robust and reproducible reporter activity were isolated. Indeed, this was found to be a successful strategy to study the tuneable mechanisms of mitochondrial regulation targeted in this study. Throughout the work, it was essential to minimise potential undesirable effects that could mask the output, e.g. toxic stress and cell death. Such effects may be caused by the treatment of interest (e.g. AICAR^19^,^29^,^30^), or by intracellular GFP accumulation^31^. Hence, the experimental conditions were established to enable long-term cell survival under repetitive stimulation/rest cycles. The subsequent analyses of cellular and mitochondrial properties confirmed that the cellular conditions and responses were of physiological relevance.

The ability of AICAR to activate AMPK, and thereby induce mitochondrial biogenesis in HeLa cells was an important condition of the present study. AICAR is known to induce phosphorylation of AMPK in an LKB1- independent manner in these cells^22^. In accordance with previous findings^32^,^33^ our data showed that AICAR activated AMPK by phosphorylation within minutes. This was accompanied by phosphorylation of ACC, which is frequently used as a marker of AMPK activity^16^. The subsequent response was a general reduction in gene transcription, which is consistent with induction of energy stress and activation of AMPK^16^. However, this effect was transient, and after the first 24 hours all markers of mitochondrial biogenesis continuously increased during the period of 6 days. Consequently, we observed increased levels of several protein subunits of the OXPHOS machinery, as well as increased rate of mitochondrial respiration. The amplification of mtDNA coincided with increased expression of TFAM, a regulator of mitochondrial transcription and mtDNA replication^34^,^35^. This was also accompanied by increased expression of NRF-1, which is known to control TFAM expression^18^. Thus, our data complements previous studies showing that AICAR activates AMPK and induces mitochondrial biogenesis via firmly established mechanisms in HeLa cells^19^,^36^, and confirms that these mechanisms are intact in the modified reporter cells. Hence, this provides rationale for using this mitoGFP-based reporter strategy to study mitochondrial mechanisms in living cells.

In cultured cells, fluorescence microscopy provides valuable opportunities to monitor both the amount and shape of mitochondria. Some cell types, such as cultured fibroblasts, may be analysed using 2D image quantification methods^24^, but for cells of a certain thickness 3D volumetric measurements are preferred^25^. Here, expression of mitoGFP reporter protein in HeLa cells facilitated mitochondrial imaging by confocal microscopy, and subsequent 3D quantification of mitochondrial biomass and morphology. Mitochondrial subpopulation analysis was established based on organelle size and shape. Our data demonstrated a close correlation between the cellular mitoGFP intensity and mitochondrial volume during treatment with AICAR. A continuum of filamentous structures forming a mitochondrial network (“reticulum”) accounted for the major fraction of the mitochondrial volume (~90% or more), in both treated and untreated cells. AICAR promoted expansion of the network, and reduced the relative amount of small organelles. Induction of mitochondrial fragmentation with CCCP increased the amounts of small organelles; however, the filamentous character remained stronger in the AICAR-treated cells. Thus, induction of mitochondrial biogenesis with AICAR was found to be associated with filament promoting and protecting effects on mitochondrial morphology.

In summary, these results provide proof of principle in support for the use of the presented mitochondrial reporter strategy to study integrated mechanisms of mitochondrial regulation and dynamics in the living cell. The results of the analysis adequately reflected effects on the innate machinery of mitochondrial biogenesis, as well as changes in mitochondrial biomass and morphology. We believe that the concepts of this work may provide useful tools to study integrated mechanisms of mitochondrial biology in a physiological context.

## Methods

### Cells and culture conditions

Phoenix A retroviral packaging cells (DR. Gary Nolan, Stanford University, USA) and HeLa cells (American Type Culture Collection, ATCC, Manassas, VA, USA) were maintained in DMEM supplemented with 10% fetal bovine serum (FBS), 2 mM L-glutamine, 100 U/ml penicillin and 100 μg/ml streptomycin (all from Sigma-Aldrich, St.Louis, MO, USA). Cells were kept in 5% CO_2_ at 37 °C.

### Cloning

The plasmids pGL3RC4/-66^37^ and pUHC 13-3 p4xNRF1^26^ with luciferase reporter sequences were a kind gift from Professor Richard Scarpulla. The promoter cassettes of these plasmids were inserted into a pEGFPmito plasmid in order to generate corresponding plasmids with mitochondrial targeted GFP reporter sequence. The pEGFPmito plasmid was initially generated by exchanging EYFP in the commercially available pEYFP-mito vector (Clontech, BD Biosciences, Franklin Lakes, New Jersey, US) with EGFP. The fluorescent protein fragments from pEGFP-N1 (Clontech, BD Biosciences) and pEYFP-mito was excised using the restriction enzymes BamHI and BsrGI and the EGFP fragment was ligated into vector with the mitochondria targeting sequence.

The promoter region containing 4 repetitions (p4xNRF1) of the NRF-1 responsive element from rat cytochrome *c* promoter was excised from pUHC 13-3 p4xNRF1 using SacI and PstI, and ligated into pEGFPmito previously cut with NheI. A negative control plasmid that lacks the NRF-1 responsive promoter region (-66mitoGFP) was constructed by excising the promoter from pGL3RC4/-66 with KpnI and NcoI. This fragment was then cloned into the pEGFPmito vector which was cut with NheI. Incompatible sticky ends were blunted when necessary. Correct orientation of the inserts was controlled using restriction enzymes HindII and NotI. All restriction enzymes in this study were purchased from Fermentas (Thermo Scientific, Waltham, MA, USA). The plasmids with correct promoter orientation were sequenced and tested in transient transfections, before cloned into the retroviral vector pTRA IRES-GFP^38^. The retroviral p4xNRF1 reporter construct was prepared using a blunt end-sticky end cloning approach. The p4xNRF1mitoGFP plasmid was first cut with BSpEI, before it was blunt-ended and cleaved with NotI. The resulting fragment was cloned into pTRA IRES-GFP, which had been opened with XhoI and blunt-ended, followed by a fragment removal of the IRES-GFP with NotI. The RC4/-66mitoGFP was cloned into the retroviral vector using XhoI-NotI restriction sites. The plasmids were sequenced to confirm correct insertion of reporter cassette. The resulting reporter plasmid is referred to as NRF1mitoGFP (Fig. 1B).

### Retroviral transduction

To generate retroviruses expressing either the NRF1mitoGFP or -66mitoGFP reporter constructs, Phoenix A packaging cells were transfected with the corresponding retroviral vector constructs by the calcium phosphate method^39^. At 6 hours post-transfection, the medium was replaced with fresh DMEM containing 10% FBS, and cells were cultured for 12 hours. The medium was then changed to the target cell medium, and the cells were grown for additionally 20 hours to produce retroviruses. Conditioned medium was collected and filtered through 0.45 μm-pore-size polysulfonic filters. Samples of these supernatants were applied immediately to the cells to be transduced, which had been trypsinised just prior to infection. Proteamine sulphate (Sigma, St. Louis, MO, USA) was added to the supernatant at a final concentration of 8 μg/ml. The cells were spin-infected by centrifugation for 90 minutes, using 1200 g and 37 °C. The cells were then incubated for 18 hours with the virus containing media. After infection, the cells were placed in fresh growth medium and cultured under routine conditions. Four days after infection, GFP positive cells were sorted using the FACS Aria (FACS-ARIA, BD Biosciences, Franklin Lakes, NJ, USA). These cells were then grown in culture for 2 weeks before further experiments were initiated.

### Establishment of inducible reporter cell clones

HeLa cells stably expressing the different reporter constructs were sorted for cells with low basal mitoGFP expression (FACS-ARIA) (Fig. 1C). These cells were further treated with 0.5 mM AICAR (Toronto Research Chemicals Inc., North York, ON, Canada) for 3 days, and cells showing high mitoGFP expression compared to untreated cells were sorted as single cells (clones) or populations (sub-lines) in 96 well plates. Following culture expansion for 4 weeks in absence of AICAR, flow cytometry analysis (Accuri™ C6, BD Accuri Cytometers Inc., Ann Arbor, MI, USA) was performed and the cultures that had returned to basal mitoGFP levels of expression were selected. Finally, AICAR-dependent induction of mitoGFP expression was confirmed after treatment with 0.5 mM AICAR for 3 days, and the clones/sub-lines with the most significant response were chosen in further experiments. In this study clone 4, referred to as HeLaNRF1/c4, were used in all experiments.

### Cell growth and treatment conditions for compound stimulation

For short term experiments (treatment up to 30 min), 0.4 × 10^6^ cells were seeded in T75 flasks one day prior to stimulation. Cells for western blotting were lysed directly in the flask. For long term experiments (up to 12 days), various number of cells were seeded in T175 flasks, depending on the length of the experiment. The cells were allowed to attach 4 hours prior to stimulation. The medium was exchanged with fresh medium with treatment after 3 days. After the given time, the cells were trypsinised in 0.25% trypsin (37 °C), counted and GFP expression was analysed by flow cytometry (Accuri™ C6). For analysis of RNA, DNA or protein, the cells were washed two times with PBS and harvested by centrifugation. The pellets were stored at −80 °C for later analysis.

### Gene expression analysis

Total RNA was extracted from cells using RNAeasy kit (Qiagen, Hilden, Germany). The resulting amount of RNA was quantified using Nanodrop 1000 Spectrophotometer (Thermo Scientific). Reverse transcription was performed from 1 μg total RNA using High-Capacity Reverse Transcription Kit (Applied Biosystems, Carlsbad, CA, US). Quantitative PCR was performed in the LightCycler 480 detection system (Roche, Basel, Switzerland) using the LightCycler 480 Probes master kit, and specific FAM probes and primers from Applied Biosystems: Nuclear respiratory factor 1; *NRF-1*, Hs00602161_m1, Transcriptional Factor A mitochondrial; *TFAM*, Hs01082775_m1 and Cytochrome C Oxidase; *Cox4i,* Hs00971639_m1. As control gene, we used 18S (RT-CKFT-18S) from Eurogentec (Liège, Belgium). Fold change was calculated by the ΔΔCt method^40^.

### Mitochondrial DNA (mtDNA) copy number

Total cellular DNA was isolated using DNAeasy blood and tissue kit and Allprep DNA/RNA Mini kit (both from Qiagen). Specific primer/probe set for mtDNA, mitochondrially encoded NADH dehydrogenase 1, ND1 (Hs02596873_s1), was purchased from Applied Biosystems and nDNA primer 18S (RT-CKFT-18S) from Eurogentec. Quantitative PCR was carried out as above and the mtDNA:nDNA ratio was calculated^40^,^41^.

### Western Blotting

Cells were washed in PBS and lysed in Ripa buffer with aprotinin (1 μg/ml), leupeptin (1 μg/ml), sodium vanadate (2 mM) and PMSF (1 mM) (all from Sigma-Aldrich). Protein concentrations were determined using BCA protein Assay (Pierce, Thermo Scientific). Proteins were separated by SDS-PAGE (10% gel), and blotted onto polyvinylidine difluoride (PVDF) membranes. The membranes were blocked with 3% BSA-TBS-T (25 mM Tris-HCl, 1mM NaCl, and 0.1%Tween 20), followed by overnight incubation with primary antibody. We employed a cocktail of 5 monoclonal antibodies against OXPHOS components (MitoProfile® Total OXPHOS Human WB Antibody Cocktail; diluted 1:500), a complex II antibody (Complex II antibody cocktail; diluted 1:500) (both from Mitosciences, Eugene, OR, USA) and antibodies against p-AMPKα and p-ACC (diluted 1:1000, Cell Signaling Technology, Inc., Beverly, MA, USA). For loading control we used β-actin antibody (Sigma-Aldrich). After immunolabelling, the membranes were washed in TBS-T and incubated with the appropriate secondary horse radish peroxidase (HRP)-conjugated antibody (Bio-Rad Laboratories, Inc., Hercules, Ca, USA). Detection was performed using the enhanced chemiluminescence kit (Bio-Rad) and the Luminescent Image analyser apparatus (Bio-Rad). Quantitative densiometric analysis of the immunoblot bands was performed using the Bio-Rad Image Lab software and the data were normalised to β-actin in each sample.

### Mitochondrial respiration

Oxygen consumption rates were studied employing an Oxygraph O2K instrument and DatLab software (Oroboros Instruments, Innsbruck, Austria), as previously described^8^. Cells were harvested, and washed in PBS, and 2 × 10^6^ cells were transferred to the 2.2 ml analytic chamber with DMEM medium. Oxygen consumption rates were then continuously monitored after sequential additions of malate (1.8 mM)/glutamate (9.1 mM), oligomycin (1.8 μg/ml), carbonylcyanide-4-(trifluoromethoxy)-phenylhydrazone (FCCP, titration to maximum activity; 0.11 μM per addition), succinate (9.1 mM)/digitonin (4.5 mg/ml, 2.5 mg per mill cells), rotenone (0.5 μM), and antimycin A (2.3 μM). Antimycin A resistant oxygen consumption was subtracted as background.

### Mitochondrial membrane potential

The cells were stained in 200 nM tetramethylrhodamine methyl ester (TMRM; Invitrogen, Carlsbad, CA, USA) for 30 minutes at 37 °C. Analysis was performed by flow cytometry (Accuri™ C6, Ann Arbor, MI, USA).

### Confocal microscopy and image analysis

For confirming mitochondrial localisation of mitoGFP, the cells were counterstained with MitoTracker Deep Red (Invitrogen). The cells were first seeded in 35 mM petri dishes with 14 mM glass bottom microwell (MatTek Corporation, Ashland, MA, USA) and allowed to adhere for 4 hours (4000 cells/dish). They were then treated with 0.5 mM AICAR for 6 days. The medium (and treatment) was replaced after 3 days. MitoTracker Deep Red (200 nM) was used as described in the manufacturer’s protocol. Images were acquired on a Zeiss LSM 510 META confocal microscope (Carl Zeiss, Oberkochen, Germany).

For quantitative 3D image analysis of mitochondrial biomass and morphology, cells were seeded (0.2 × 10^6^ cells/T75 flask) and allowed to adhere overnight. They were then treated with 0.5 mM AICAR for 6 days; including medium and treatment replacement after 3 days. On day 5, the cells were reseeded to a suitable density (0.5–1 × 10^5^ cells/chamber) in microscopy chamber slides (Lab-Tek™ II Chamber Slides, 2 or 4 chambers/slide, Thermo Scientific). The original medium was collected before the cells were trypsinised (0.25% trypsin, 37 °C), and transferred to the corresponding reseeded cultures. On day 6, the medium was replaced with microscopy medium (pH7.4) consisting of DMEM (phenolred-free) supplemented with 10 mM glucose, 2 mM l-glutamine, 2 mM sodium pyruvate and 5 mM HEPES (all from Sigma). Control cells were cultured without the presence of AICAR. For the mitochondrial fragmentation analysis CCCP (final concentration of 5 μM) was added 30–60 min before imaging.

Confocal microscopy of mitoGFP was performed on a Zeiss LSM 510 META confocal microscope using a Plan-Apochromat 63 × 1.40 NA oil objective. Image acquisition, processing and analysis were performed essentially as described thoroughly in^25^, using the Image-Pro Plus software (version 7.0) (Media Cybernetics, Inc., Washington, USA). An illustration of the consecutive steps is shown in Fig. 3A. The acquired 12 bit z-stacks were background corrected (fixed level within each experiment), and processed by 3D blind deconvolution. In order to enable single cell analysis, each z-stack, which in general contained 2–5 cells, was manually masked to produce z-stacks with single cells. These z-stacks were processed with the 3D open filter (x, y, z: 3, 3, 3), followed by loading into the 3D module of the software. Volumetric models of the mitochondria were constructed using no subsampling or simplification settings. The surface level was fixed for the experiments in this study. Objects larger than 0.05 μm^3^ were accounted as mitochondrial objects. For measurement of mitoGFP intensity in the mitochondrial compartment, the 3D isosurface created from the processed z-stacks were applied as mask on the corresponding z-stacks that had not been processed by the 3D open filter.

### Statistical analysis

Statistical analysis was performed using a standard two-sided Student’s t-Test (where not indicated otherwise), and one-way ANOVA with Tukey’s multiple comparisons test. Differences with p < 0.05 were considered as significantly different. Three individual experiments were performed if not indicated otherwise.

## Additional Information

**How to cite this article**: Nilsson, L. I. H. *et al.* A new live-cell reporter strategy to simultaneously monitor mitochondrial biogenesis and morphology. *Sci. Rep.* **5**, 17217; doi: 10.1038/srep17217 (2015).
