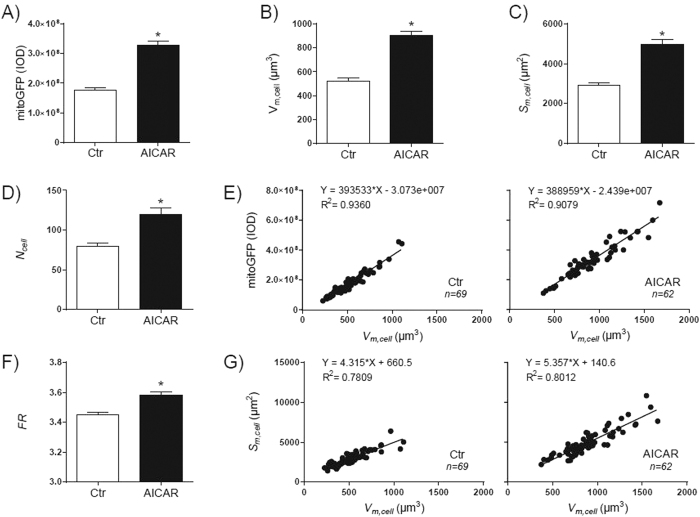# Corrigendum: A new live-cell reporter strategy to simultaneously monitor mitochondrial biogenesis and morphology

**DOI:** 10.1038/srep30527

**Published:** 2016-08-19

**Authors:** Linn Iren Hodneland Nilsson, Ina Katrine Nitschke Pettersen, Julie Nikolaisen, David Micklem, Hege Avsnes Dale, Gro Vatne Røsland, James Lorens, Karl Johan Tronstad

Scientific Reports 5: Article number: 1721710.1038/srep17217; published online: 11 24 2015; updated: 08 19 2016.

This Article contains errors in Figure 4.

In Figure 4B, the y-axis ‘V_*m,cell*_ (µm^3^)’ is incorrectly labelled as ‘V_*m,cell*_ (m^3^)’.

In Figure 4C, the y-axis ‘*S*_*m,cell*_ (µm^2^)’ is incorrectly labelled as ‘*S*_*m,cell*_ (m^2^)’.

In Figure 4E, the x-axes ‘V_*m,cell*_ (µm^3^)’ are incorrectly labelled ‘V_*m,cell*_ (m^3^)’.

In Figure 4G, the x-axes ‘V_*m,cell*_ (µm^3^)’ and y-axes ‘*S_m,cell_* (µm^2^)’ are incorrectly labeled as ‘V_*m,cell*_ (m^3^)’ and ‘*S*_*m,cell*_ (m^2^)’ respectively.

The correct Figure 4 appears below as Figure 1.

## Figures and Tables

**Figure 1:**